# Reducing Fear to Help Build Healthy Families: Investing in Non-Punitive Approaches to Helping People with Substance Use Disorder

**DOI:** 10.1007/s10995-023-03772-8

**Published:** 2023-09-27

**Authors:** Karen A. Scott, Maridee Shogren, Kenneth Shatzkes

**Affiliations:** 1 Foundation for Opioid Response Efforts, 110 West. 40th Street, New York, NY 10018 USA; 2 College of Nursing and Professional Disciplines, University of North Dakota, 430 Oxford Street Stop 9025 Grand Forks, Grand Forks, ND 58202 USA

**Keywords:** Opioids, Maternal mortality, Philanthropy, Treatment, Stigma, Child welfare reporting

## Abstract

**Background:**

Many pregnant and parenting people with substance use disorders (SUD) refrain from seeking perinatal care or treatment for their SUD for fear of being treated poorly by health care providers and/or triggering a child welfare investigation. For those who do seek treatment, there are relatively few clinicians willing and able to prescribe medications for opioid use disorder (MOUD) to pregnant people. Both stigma and lack of access to treatment put many pregnant and parenting people at risk. Drug-related deaths contribute significantly to U.S. maternal mortality rates, with people at especially high risk of drug overdose in the months following delivery.

**Methods:**

The Foundation for Opioid Response Efforts (FORE) is a national philanthropy focused on finding and fostering solutions to the opioid crisis. We draw lessons from our grantees’ efforts to expand access to substance use treatment and recovery supports for pregnant and parenting people.

**Results:**

To build systems of care that ensure more pregnant people get timely perinatal care, we need to expand training for perinatal providers on how to provide OUD treatment, clarify child welfare reporting rules, and engage and support trusted organizations and community-based services.

**Conclusions:**

In addition to changes to our systems of SUD treatment and recovery, we need greater philanthropic investment in efforts to combat the public health crisis of substance use and overdose among pregnant and parenting people. Private funders have the leeway to act quickly, take risks, and demonstrate the effectiveness of new approaches, building the case for investment of public resources in such initiatives.

## Significance

The continuing high rates of overdose deaths in the U.S. intersect with maternal mortality and morbidity, as well as family health. Private foundations along with public agencies have significant opportunities to align work on both issues to save lives.

A recent episode of *Call the Midwife*, set in the early 1960s in East London, could be mistaken for the present day. A young pregnant woman with a heroin addiction was terrified to seek prenatal care because she feared health care staff would discover her drug use and take her baby away. After skipping her prenatal care appointments, she went into early labor and delivered a low-birthweight infant who then suffered from opioid withdrawal. The mother was offered medication but worried that taking it would jeopardize the chances of keeping her child. Raised by a mother with alcohol use disorder, she had already experienced this trauma when she was placed in foster care. While the language of the time — such as “the baby’s addicted” — reflects outdated ideas, the plot could have happened yesterday.

Sixty years later, pregnancy, substance use disorder (SUD), and its sequelae remain shrouded in fears that can limit access to evidence-based treatment for both prenatal and SUD care, increase the risk of complications or deaths, and lead to family separations.

Although most forms of medications for opioid use disorder (MOUD), the gold standard for treatment of opioid use disorder (OUD), are safe to administer during pregnancy, relatively few clinicians are willing and able to prescribe MOUD to pregnant people (Titus-Glover et al., 2021; White House Office of National Drug Control Policy, 2022). As with many aspects of health care access, racial disparities in perinatal OUD treatment have also been documented, with Black and Hispanic women less likely to have received MOUD than white women during the perinatal period (Schiff et al., 2020; Short et al., 2018). State child welfare reporting laws further instill fear in pregnant people and often generate confusion among the providers caring for them (Roberts et al., 2022).

Meanwhile, maternal outcomes in the United States worsen. The latest *Health of Women and Children Report* from America’s Health Rankings shows that, between 2019 and 2020, mortality rates increased 21% among women ages 20–44 while severe maternal morbidity increased by 5% between 2018 and 2019 (United Health Foundation, 2022). The report also shows significant racial disparities in data between 2016 and 2020: while there were 19.3 maternal deaths per 100,000 live births across all pregnancies, among Black and American Indian/Alaska Native American mothers the rate was at least twice as high (52 deaths per 100,000 live births for Black mothers and 39.4 deaths per 100,000 live births for American Indian/Alaska Native mothers). Drug-related deaths significantly contribute to these mortality rates. A recently published analysis found that drug-related deaths accounted for slightly more than 11% of pregnancy-associated deaths between 2010 and 2018 and increased 190% over this time period (Margerison et al., 2022). In some states, including Ohio and Tennessee, overdose is the leading cause of pregnancy-related death in the year following delivery (White House Office of National Drug Control Policy, 2022).

The Foundation for Opioid Response Efforts (FORE) is a national grantmaking philanthropy launched in 2018 to find and foster solutions to the opioid crisis. Our programmatic work focuses on: increasing access to treatment and recovery services; promoting innovation that generates timely and actionable data on the overdose crisis as well efforts to reduce stigma; and expanding access to family- and community-based prevention programs in schools and other settings. We prioritize programs and initiatives geared toward populations at highest risk for overdose and OUD morbidity. This includes pregnant and parenting people, who face much higher risk of overdose death in the several months following delivery (Massachusetts Department of Public Health, 2017). To date, we have awarded more than $38 million in grants to 98 organizations around the United States. Our grantees include researchers, advocates, and clinicians working in academic, health care, and research institutions as well as in community-based organizations that are on the frontlines of the opioid crisis. Through our convening activities, we engage with many others, including colleagues in the public sector and people with lived experiences of SUD. Additionally, we develop resources to share lessons and tools from our grantees with private- and public-sector leaders who can spread promising approaches.

A recent convening of FORE’s grantees included a session on the current environment for reproductive health and SUD. Three of FORE’s grantees — Hendrée Jones, Ph.D., of the University of North Carolina at Chapel Hill (UNC, UNC Horizons); Mishka Terplan, M.D., M.P.H., of Friends Research Institute (FRI); and Maridee Shogren, D.N.P., C.N.M., of the University of North Dakota (UND) — discussed the challenges faced by people with SUDs who are seeking pregnancy and postpartum services. The first word that came up was “fear.” Many pregnant people with SUDs are afraid of being stigmatized and treated poorly by health care providers. Many also worry about being separated from and potentially losing their children to the foster care system. Such fears have been exacerbated by the *Dobbs* decision, since some people have lost the ability to decide whether it is the right time for a pregnancy. Providers, too, worry about balancing their duties to patients against their legal requirements. Despite this climate of fear, there are promising initiatives pointing the way forward.

The FORE-funded work at UND is one such example. UND launched “Don’t Quit the Quit” (DQTQ) in 2020 to expand access to OUD treatment during pregnancy and help people stay in treatment postpartum. In 2021, DQTQ was recognized by North Dakota’s governor for its contributions to improving recovery services in the state.

Thus far, the DQTQ program has trained 13 health care providers, including family practice physicians, physician assistants, and nurse practitioners who offer obstetric care, to provide MOUD. This has increased access to MOUD in 12 of North Dakota’s most rural counties, including two Tribal Nations. Four faculty members from UND’s family medicine residency program also participated in the training as they prepared to open a MOUD clinic that will expand access to treatment while educating residents on how to prescribe MOUD.

DQTQ has also been recruiting and training community members to serve as postpartum doulas and support rural families affected by OUD. Postpartum doulas routinely offer supports to new mothers, newborns, and other family members. However, their training does not typically include information about SUDs or the role doulas can play in increasing awareness of SUD and helping families find treatment and other support. The DQTQ program incorporated these elements into its doula training.

The DQTQ program has also been training staff from North Dakota’s Women, Infants, and Children (WIC) Program about SUD, OUD, and treatment options. WIC agencies are well suited to increase awareness of SUD during the perinatal period. Staff are already required to: screen all participants for substance use and/or environmental tobacco smoke exposure; provide participants with education at each certification; increase participants’ access to information about the dangers of substance use during pregnancy and breastfeeding; and facilitate referrals for further assessment as appropriate (U.S. Department of Agriculture, 2013). And while these requirements exist, DQTQ is a first-of-its-kind training program to provide WIC staff with the knowledge and tools to be able to help participantswith OUD. While as many as 40% of people do not attend a postpartum medical visit (McKinney et al., 2018), WIC staff tend to see people frequently during the postpartum period, when they come in for health screenings and food supports.

The DQTQ program worked with WIC agencies in 12 rural North Dakota counties to provide staff with the tools to screen for SUD, offer a brief intervention to educate families about SUD, and discuss referrals, if needed. Ongoing education about stigma reduction and the development of an infographic appropriate for WIC clients has enhanced support for all North Dakota families who seek WIC services. The DQTQ program has since expanded this training to WIC agencies in several other states and has presented on this topic to the National WIC Association and the National Indigenous and Native American WIC Coalition. In fulfilling educational requests from WIC agencies over several years, our grantees have found the most frequently requested topic is reducing stigma toward the perinatal population.

By working closely with FORE grantees like the DQTQ program and others across the U.S., we are continually learning how to build systems of care that promote healthy families and ensure more pregnant people get timely perinatal care. We’ve learned the framework for building welcoming and accessible systems for those seeking treatment and supports depends on foundational elements including:

- **Expanding OUD training for perinatal providers.** Having more options to access OUD treatment and more providers trained to deliver it would help to normalize this care. The White House Office of National Drug Control Policy’s, 2022 report on substance use and pregnancy notes that only 10% of obstetrician-gynecologists and midwives were waivered to prescribe the MOUD buprenorphine. The OMNIBUS legislation passed at the end of 2022 eliminated the need for this waiver. While this change has the potential to significantly increase the number of clinicians who will treat OUD, treatment is still a new area of clinical practice for many providers. In FORE-funded programs, we have seen that education, training, and coaching are important mechanisms to expand the number of clinicians offering OUD treatment (Shatzkes & Scott, 2021). For example, investing in robust training that provides opportunities for clinicians to review cases with other clinicians experienced in the field of perinatal SUD, in as close to real time as possible, could strengthen treatment capacity.
- **Engaging and supporting trusted organizations and community-based services.** Offering social, emotional, and other supports can help pregnant and parenting people access substance use treatment; improve the health of parents and their children; and promote strong families. These supports may include peer recovery specialists, or those with lived experiences of SUD who can provide a deep level of understanding of people’s concerns and challenges. Additionally, supporting novel approaches, such as training postpartum doulas and WIC staff, can leverage the existing resources of a community in new ways. Another of our grantees, UNC Horizons, launched a perinatal SUD program for women in a county jail. Staff meet women as they leave incarceration. In addition to linking them with wraparound supports, they ensure there is no disruption in their treatment. The state of North Carolina is now providing funds to continue and expand this program. Further evaluation of these models is needed, as is sustainable reimbursement.
- **Clarifying child welfare reporting rules.** Fear and confusion regarding child welfare reporting can dissuade people from seeking health care either for their pregnancy or their substance use, increasing risks of pregnancy-related complications and OUD-related deaths. FORE grantees Terplan and Sarah Roberts, Dr.P.H., at the University of California, San Francisco, are developing, implementing, and evaluating trainings for health care providers, hospital administrators, and public health officials to clarify reporting requirements, as well as what is not required. The goal is to move health care professionals away from what is often a reflexive, punitive reporting stance toward a more considered approach.

Regulations that require urine drug testing at the time of delivery — often without patient consent — can also discourage pregnant people from seeking OUD treatment, even when that may be the safest option for both parent and baby. A recent analysis found that Black patients giving birth at one health system were more likely to be tested for drug use than white people and less likely to test positive (Jarlenski et al., 2023). A FORE-funded project with Montefiore Medical Center in New York City is developing and disseminating two evidence-based toolkits; one will guide patient–physician decision-making on whether, when, and how to use urine drug testing and the other will inform people of their rights related to drug testing. While clarifying current rules is important, evaluating their impact and identifying reforms that might lead to better outcomes for the whole family are also very much needed.

We see these as foundational steps in building a coordinated system of care that places perinatal people at the center; recognizes the clinical, social, and legal challenges they may face; and works toward the goal of building healthy, resilient families. This must begin by acknowledging and addressing the fears that prevent people with OUD from seeking treatment, as well as the impediments health care and community service providers face in offering supports.

Advancing this work also requires greater and more sustained investment from philanthropy. Private funders have the leeway to act quickly, take risks, and demonstrate effectiveness, building the case for investment of public resources in such initiatives. Given the intersecting medical, legal, and social issues presented in addressing substance use among pregnant and parenting people, we see opportunities for private philanthropies to work proactively with one another and in collaboration with public sector agencies. Based on FORE’s experience, we offer the following recommendations to funders:

- **Find common ground.** SUD care is often siloed from other medical specialties, including maternal, child, and behavioral health, making it difficult to advance the comprehensive, integrated care that it often needed for this population. Philanthrophy can be similarly siloed, but what many in philanthropy share is a focus on populations at greatest risk or vulnerability.
  - Funders should look for opportunities to break down these siloes and fund work that fosters collaboration.
  - Funders can also look to partner with peers in different sectors. For example, a foundation focused on early childhood development can find common ground and complement the work supported by a funder focused on SUD treatment for a parent. Both are needed to achieve the best outcomes.
- **Consider longer funding cycles.** Funding pilot efforts for longer periods of time can help ensure grantees have sufficient time to demonstrate efficacy and impact. As an example, FORE extended the timeline of grants in its Prevention program to ensure grantees had the time to demonstrate the benefit of their work.
- **Support policy work for long-term sustainability and impact.** As public funding does not often fund initiatives that elucidate gaps in policy and regulation, private funders could play a larger role in supporting policy research and advocacy that identifies in the impact of policy on care systems and treatment outcomes.
- **Building capacity and partnerships.** In soliciting proposals, funders could incentivize or require partnerships between different community entities, particularly with community-based organizations. For example, FORE’s Innovation program sought to fund projects that brought together teams from different sectors to address intractable problems that impede efforts to end the opioid and overdose crisis. FORE’s Community-Driven Responses program acts to build the capacity of local organizations by providing funding for general operating costs.

A strong, concerted effort from philanthropy will also help reduce stigma against addiction, which will help to counteract the fear the pregnant and parenting people face in seeking help. We at FORE commend all efforts to support people with an SUD, as well as their families and their providers, and call on private foundations to join in partnership with other funders as well as public agencies and other organizations. Together we can expand access to SUD treatment and recovery services throughout the perinatal period to address this multigenerational public health crisis and save lives.

## Data Availability

N/A.

## Code Availability

N/A.
